# *Drosophila* Duplication Hotspots Are Associated with Late-Replicating Regions of the Genome

**DOI:** 10.1371/journal.pgen.1002340

**Published:** 2011-11-03

**Authors:** Margarida Cardoso-Moreira, J. J. Emerson, Andrew G. Clark, Manyuan Long

**Affiliations:** 1 Department of Molecular Biology and Genetics, Cornell University, Ithaca, New York, United States of America; 2 Department of Integrative Biology, University of California Berkeley, Berkeley, California, United States of America; 3 Department of Ecology and Evolution, University of Chicago, Chicago, Illinois, United States of America; Fred Hutchinson Cancer Research Center, United States of America

## Abstract

Duplications play a significant role in both extremes of the phenotypic spectrum of newly arising mutations: they can have severe deleterious effects (e.g. duplications underlie a variety of diseases) but can also be highly advantageous. The phenotypic potential of newly arisen duplications has stimulated wide interest in both the mutational and selective processes shaping these variants in the genome. Here we take advantage of the *Drosophila simulans*–*Drosophila melanogaster* genetic system to further our understanding of both processes. Regarding mutational processes, the study of two closely related species allows investigation of the potential existence of shared duplication hotspots, and the similarities and differences between the two genomes can be used to dissect its underlying causes. Regarding selection, the difference in the effective population size between the two species can be leveraged to ask questions about the strength of selection acting on different classes of duplications. In this study, we conducted a survey of duplication polymorphisms in 14 different lines of *D. simulans* using tiling microarrays and combined it with an analogous survey for the *D. melanogaster* genome. By integrating the two datasets, we identified duplication hotspots conserved between the two species. However, unlike the duplication hotspots identified in mammalian genomes, *Drosophila* duplication hotspots are not associated with sequences of high sequence identity capable of mediating non-allelic homologous recombination. Instead, *Drosophila* duplication hotspots are associated with late-replicating regions of the genome, suggesting a link between DNA replication and duplication rates. We also found evidence supporting a higher effectiveness of selection on duplications in *D. simulans* than in *D. melanogaster*. This is also true for duplications segregating at high frequency, where we find evidence in *D. simulans* that a sizeable fraction of these mutations is being driven to fixation by positive selection.

## Author Summary

DNA duplications are important contributors to the phenotypic differences observed between individuals. These mutations can disrupt the normal functioning of genes and so are often associated with disease. But because they can add genetic information they can also lead to evolutionary change. Understanding how selection and non-random mutation processes shape the distribution of duplications throughout the genome is important to elucidate both the medical and evolutionary impacts of these mutations. Here, we examined the roles of selection and mutation in shaping patterns of duplication polymorphisms across the genomes of the fruit fly *Drosophila melanogaster* and its sister species, *D. simulans*. We found that selection is pervasive in both genomes but is more efficient in *D. simulans* than in *D. melanogaster*. We also found that these two species have shared duplication hotspots, i.e. orthologous regions experiencing high rates of duplication in the two genomes. After excluding the hypothesis that *Drosophila* duplication hotspots are associated with regions of the genome rich in segmental duplications (as observed for mammalian genomes), we show that they are associated with late-replicating regions of the genome. Our work therefore proposes a link between DNA replication and rates of duplication across the genome.

## Introduction

In 2004, two pioneering studies showing that copy number variants (CNVs) are abundant in healthy human individuals [1], [2] accelerated research on this class of variation. The focus on these variants was well motivated because duplications and deletions of DNA regions have long been known to underlie a variety of genomic disorders [3], [4]. The discovery of the abundance of CNVs in otherwise healthy individuals made them good candidates to underlie common and rare diseases as well as other physiological traits. In just a few years, CNVs were implicated in a variety of diseases such as autism [5], schizophrenia [6], Crohn's disease [7], psoriasis [8] and other traits such as body weight [9] and starch consumption [10]. Duplications and deletions also have a long history of being implicated in adaptation and of being a major source of genetic innovation [11]–[14]. In domesticated animals, for example, they are responsible for white coat color in horses (duplication within an intron leading to *cis*-regulatory changes [15]), reduced comb and wattle size in chickens (duplication within an intron leading to expression changes [16]) and short-legged dogs (new retrogene [17]). Although much has been learned about CNVs, recent research raises more questions than it answers. Two independent avenues of research focus on studying the roles played by mutation and selection on copy number variation.

Understanding the mutational processes underlying the formation of CNVs is important from both a medical and an evolutionary perspective. Duplications and deletions can result from the imperfect repair of DNA double strand breaks generated by both exogenous (e.g. ionizing radiation) and endogenous (e.g. reactive oxygen species) agents as a consequence of the normal cellular metabolism [18], [19]. DNA replication errors can also generate CNVs, with or without the formation of DNA double strand breaks [4], [19]. Replication-based repair processes have been proposed to explain complex CNVs (i.e. CNVs with multiple breakpoints) [20]–[22] but evidence suggests they underlie the formation of simple CNVs as well [23]–[25]. Several lines of evidence suggest that CNV mutation rates vary throughout the genome [26], [27] and CNV hotspots have been identified in the human [27]–[29], chimpanzee [28], [30], mouse [31] and fly [32]–[34] genomes. Mammalian CNV hotspots are significantly enriched with segmental duplications, which have been proposed to promote the occurrence of CNVs by facilitating non-allelic homologous recombination (NAHR) [3], [4]. Following this observation, Sharp and colleagues specifically targeted genomic regions associated with segmental duplications in the human genome and were able to identify CNVs associated with previously unidentified genomic disorders [35]. But not all mammalian hotspots are associated with segmental duplications [28], [30] and *Drosophila* hotspots are likely not associated with them at all [33]. As such, a priority of the field is to identify the genomic feature(s), other than segmental duplications, that are associated with regions with increased numbers of CNVs.

Understanding the evolutionary forces shaping the evolution of CNVs is also important from a medical and evolutionary perspective. Despite their pervasiveness, analyses of the genomic distribution of CNVs among different functional regions clearly indicate that a large fraction is under purifying selection. Population genetic models that address both demographic and selection processes have been used to estimate the strength of selection acting on different classes of CNVs. In both flies [36] and humans [37] coding CNVs are under the strongest purifying selection followed by intronic CNVs and finally intergenic CNVs. Evidence for positive selection has been less clear. There are examples of CNVs under positive selection in humans, such as the copy number variation of the amylase [10] and CCL3L1 [38] genes, and in flies (e.g. duplication of the Cyp6G1 locus) [36], [39]. However, on a genome-wide scale, the over-representation of certain classes of genes in CNVs, namely “environmental” genes, is best explained by reduced purifying selection acting on these variants than by positive selection [40]. Although genome-scale studies of CNVs have only recently become technically feasible [41], the study of gene duplication can be traced back to as early as 1911 [12], [42]. An important problem is to determine the relative roles of positive selection and genetic drift in the fixation of new gene duplicates [43]. Most population genetic models assume that gene duplicates are fixed by genetic drift and that their subsequent fate in genomes (being retained or lost) is determined by ensuing mutations in one or both copies [43], [44]. An alternative hypothesis is that gene duplications are fixed by positive selection. Assessing the roles of drift and selection requires the study of young duplications that still bear the hallmarks of the evolutionary process responsible for their fixation [11], [14], [43].

The aim of this work is to investigate the roles played by mutation and selection on duplication polymorphisms. We take advantage of the genetic model system composed by the sibling species *D. melanogaster* and *D. simulans*, which have been used extensively to conduct population and evolutionary genetic studies [45]. While they share a recent common ancestor and are morphologically very similar, at an average of 4% DNA sequence divergence, they are sufficiently diverged to provide many evolutionary insights [46]. Hence, the structural differences (and similarities) of their genomes can be leveraged to dissect the genomic features responsible for the variation in CNV density along the genome and elucidate the existence of duplication hotspots. For example, while the *D. melanogaster* genome is rich in inversion polymorphisms these are rare in *D. simulans* [47]. Similarly, the fraction of repetitive sequence is considerably larger in the *D. melanogaster* genome [46], [48] and transposable elements are differentially distributed in the two species [46]. Another useful distinction between the two species, and one that can be used to investigate the role of selection, is the difference in their effective population sizes. *D. simulans* has a ten-fold larger effective population size than *D. melanogaster*, which is predicted to translate into a greater effectiveness of selection in *D. simulans* [49], [50]. Thus this species is expected to be more efficient both at purging deleterious mutations and fixing those that are beneficial [51]. The differences in population size and genome structure between *D. melanogaster* and *D. simulans* provide us with a powerful genetic model in which to study how mutation and selection processes shape patterns of copy number variation.

Duplication and deletion polymorphisms have previously been surveyed in 15 lines of *D. melanogaster* using tiling microarrays [36]. Here, we use the same approach to identify and characterize duplication polymorphisms in 14 lines of *D. simulans*. By integrating this new dataset of duplications in *D. simulans* with the previous dataset of duplications in *D. melanogaster*, we identified duplication hotspots shared between the two species. Significantly, we found that these hotspots are not associated with segmental duplications or transposable elements but are instead associated with regions of the genome that are late-replicating. We also show a higher effectiveness of selection acting on *D. simulans* duplications than on *D. melanogaster* duplications, and suggest an important role for positive selection in driving a sizeable fraction of *D. simulans* duplications to fixation.

## Results

### A snapshot of duplication polymorphisms in the *D. simulans* genome

We identified polymorphic duplications in the *D. simulans* genome using a similar strategy to the one previously used to identify CNVs in the *D. melanogaster* genome [36]. Briefly, we hybridized DNA from 14 *D. simulans* lines (see Materials and Methods) onto DNA tiling arrays (three replicates per line). Because the tiling arrays were designed based on the *D. melanogaster* genome, of the ∼3 million probes available on the arrays, only the ∼900,000 that had a perfect (and unique) match to the *D. simulans* genome were used in this study (see Materials and Methods). The hybridization intensities were then decoded into probabilities of copy number gains and losses (and absence of changes in copy number) using a Hidden Markov Model. A given region was called as a putative duplication if at least two consecutive probes gave hybridization signals decoded as having at least a 40% posterior probability of being duplicated.

We identified 830 duplications segregating in the 14 *D. simulans* lines (Table S1). The duplications are on average 1.8 kb in size (median 424 bp), with the smallest being 28 bp and the largest ∼127 kb. We evaluated the quality of the CNV calls by PCR. Our PCR assay assumed duplications occur in tandem, such that a pair of divergent primers placed within the region predicted to be duplicated would lead to the amplification of a band only in the presence of a tandem duplication. Sequencing of that band would provide the exact duplication breakpoints. Out of 24 putative duplications, 18 produced the expected band, yielding a confirmation rate of 75%. The remaining 6 duplication candidates yielded no band, which could suggest: 1) the duplication is a false positive; 2) the duplication is not in tandem; or 3) the PCR reaction failed. To exclude the third possibility, we designed pairs of convergent primers (outside the putative duplication) for the 6 unconfirmed duplications, such that lines predicted to have a duplication would produce larger bands than the lines without it. This strategy confirmed one of the 6 remaining duplications, increasing the confirmation rate to 79%. A survey of recently fixed gene duplications in *Drosophila* [52], found that 82% of duplications in *D. melanogaster* and 78% in *D. yakuba* occur in tandem, with the remaining being dispersed in the genome. Our confirmation rate is, therefore, in good agreement with the expectation for the proportion of tandem duplications, and further supports the view that the majority (∼80%) of newly generated duplications occur in tandem, with the remaining being dispersed throughout the genome (including at a certain distance from each other within the same chromosome). It is important to note that since our confirmation strategy involved designing primers within the predicted duplication, we only attempted to confirm duplications larger than 300 bp (see Materials and Methods).

We also detected 379 deletions. However, out of 32 deletions assayed by PCR (primers located outside the putative deletion), only 13 were confirmed, yielding a false positive rate of almost 60%. The Sanger sequencing of the false positives revealed the presence of small indels and SNPs overlapping with the probes that were called as being deleted. Because the probes in the tiling arrays are only 25 bp, any variant that occurs within them knocks out the hybridization signal in a manner similar to a deletion. This same problem was encountered when characterizing deletions in *D. melanogaster* (false positive rate of 47% [36]). Because most of our deletion calls are likely to be false positives, we did not include these variants in our study.

Distinguishing novel duplications segregating in the lines examined (derived duplications) from ancestral duplications is important. The latter correspond to situations where the duplication is fixed in *D. simulans* but because it is present in the reference genome sequence as a single copy (due to deletion or genome mis-assembly) it appears in our survey as a duplication segregating at high frequency. Distinguishing derived from ancestral duplications can be accomplished by determining the duplication status of these regions in the *D. melanogaster* genome. Derived duplications would appear as single copy regions in both the *D. melanogaster* and *D. simulans* reference genomes, whereas ancestral duplications would appear as duplications in *D. melanogaster*. We found only one event for which there was evidence of a duplicate in the *D. melanogaster* genome reference sequence. However, we found three duplications in *D. simulans* that appear in the *D. melanogaster* genome reference sequence as single-copy, but are detected as polymorphic duplications in all (or most) of the 15 lines previously used to identify CNVs in the *D. melanogaster* genome [36]. This result is not entirely surprising given the nature of the ascertainment bias when inferences are made from arrays designed from a single reference sequence [36], [53]. Two of these duplications are predicted to have identical breakpoints in the two species and are detected in all lines in both species. The third duplication, completely encompasses two genes involved in drug metabolism (Ugt86Dj and Ugt86Dh), and was detected in all 14 *D. simulans* lines and in 13 out of the 15 *D. melanogaster* lines. We sequenced the breakpoints of this duplication in both species and they are identical, suggesting they derive from the same mutation. The most unusual aspect of these events is their apparent absence in the genome references, which should be unlikely if the duplication is ancestral to both *D. melanogaster* and *D. simulans*. The most likely explanation is that the duplicates became fixed before the split of *D. melanogaster* and *D. simulans* and were either collapsed during the genome assemblies or the sequenced genome strains contain deletions of one of the copies. For the third duplication mentioned, given that a cost of resistance can be associated with insecticide resistance [e.g. 54] it is perhaps not surprising that strains shielded from the selective pressure of insecticides may preferentially lose such mutations under laboratory cultivation.

Although duplications are found throughout the whole genome, they are distinctly less frequent in functional elements: even though 41% of the *D. simulans* genome is annotated as coding sequence [55], only 28% of duplications overlap with these regions. The majority of duplications are restricted to intergenic (50%) and intronic (22%) regions, implying that a large fraction of these mutations are deleterious and are quickly removed from the populations by purifying selection. Overall, duplications are kept at very low frequencies in the lines surveyed, with 83% of them being detected in only one of the 14 lines (Table S1).

**Figure 1:**
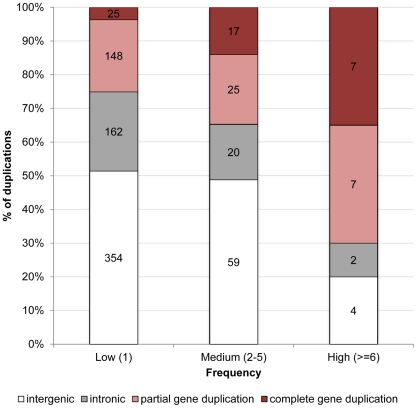
Proportion of duplications in low-, medium-, and high-frequency overlapping different genomic contexts in *D. simulans*. A duplication is said to be intergenic if it overlaps exclusively intergenic sequence, to be intronic if it overlaps exclusively intronic sequence, to be a partial gene duplication if it encompasses exonic or exonic and intronic sequence, and to be a complete duplication if it encompasses a complete gene structure. The numbers in the columns refer to the number of duplications observed in each class.

Figure 1 illustrates that the distribution of duplications among genomic contexts varies dramatically between those that are kept at very low frequencies, e.g. singletons (1 out of 14 lines), and those that are segregating at high frequencies (found in at least 6 of the 14 lines). Counterintuitively, while only 25% of duplications segregating at very low frequencies overlap coding sequence (i.e. partial and complete gene duplications), 70% of duplications segregating at high frequencies encompass coding sequence (Fisher exact test, p = 0.0001). If genetic drift was responsible for high frequency derived alleles, one would not predict such duplications to overlap coding sequence, because these mutations are less likely to be neutral [36]. This apparent contradiction can be resolved if we instead posit that positive selection plays an important role in driving these mutations to fixation. In support of this hypothesis, we found that while complete gene duplications represent 3.6% of duplications segregating in only one line, they represent 35% of duplications segregating in 6 or more lines (Fisher exact test, p = 9.95×10^−6^). Although there is also an increase in the proportion of partial gene duplications (from 22% of all duplications segregating in only one line to 35% of duplications segregating in 6 or more lines), this increase is not statistically significant (Fisher exact test, p = 0.2). This means that of duplications overlapping exonic sequence, only complete gene duplications are over-represented among high-frequency variants. There is no Gene Ontology category over-represented in the set of genes present in high-frequency duplications (p>0.01).

### Comparison of patterns of duplication polymorphism between *D. simulans* and *D. melanogaster*

In a previous study, Emerson and colleagues used the same microarray platform and a similar strategy to detect duplication polymorphisms in 15 *D. melanogaster* lines [36]. In that study all ∼3 million probes present in the tiling arrays were used to make the duplication calls (as opposed to the ∼900,000 available for this study) which provided more power to detect them (especially the smaller ones) and better breakpoint resolution. As expected, more duplications were detected in *D. melanogaster* than in *D. simulans* (2016 vs. 830), and the former were also, on average, shorter (1.2 kb vs. 1.8 kb) although the difference is not statistically significant (Wilcoxon rank sum test, p = 0.8). The set of *D. melanogaster* duplications used here differs from the set originally published by the exclusion of those duplications detected by only one probe because these were not included in the set of *D. simulans* duplications. Figure S1 shows the genomic location of the duplications detected in both species.

**Figure 2:**
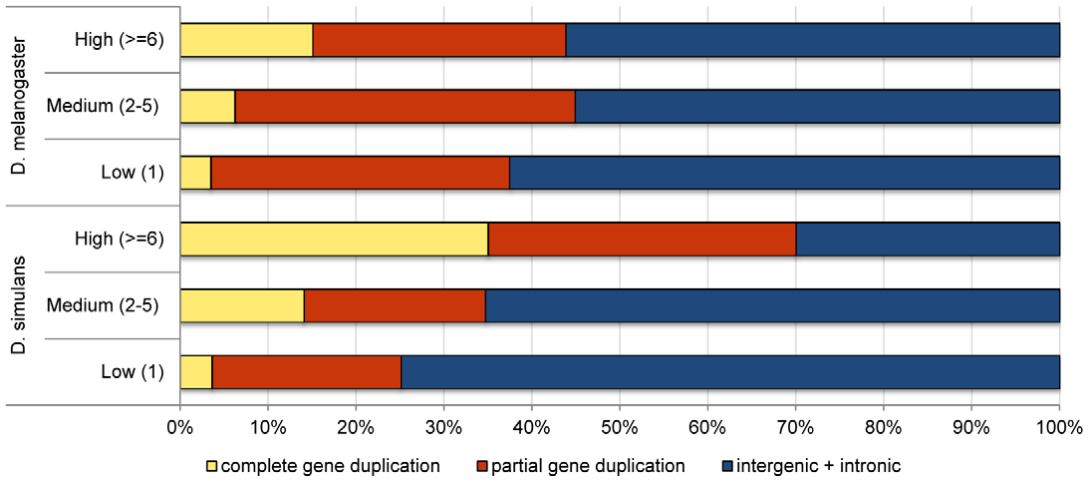
Comparison of the proportion of duplications in low-, medium-, and high-frequency overlapping different genomic contexts in *D. simulans* and *D. melanogaster*.

Although a higher proportion of the *D. simulans* genome is annotated as coding (41% vs. 33% in *D. melanogaster*) [55], we found that *D. simulans* has a significantly lower proportion of coding duplications than *D. melanogaster* (28% in *D. simulans* vs. 39% in *D. melanogaster*, Fisher exact test, p = 2×10^−9^), suggesting purifying selection acts more strongly on *D. simulans* duplications. Figure 2 shows, for the two species, the proportion of the different classes of duplications partitioned by their frequency in the lines surveyed. While *D. simulans* has a smaller proportion of partial and complete gene duplications segregating in low and medium frequencies than *D. melanogaster* (consistent with stronger purifying selection), the opposite pattern is observed for high frequency duplications. In this latter class, *D. simulans* has a significantly higher proportion of complete gene duplications than *D. melanogaster* (35% vs. 15%, respectively; Fisher exact test, p = 0.0001). Because complete gene duplications are more likely to be advantageous than all other classes of duplications, this result can be interpreted as supporting a more pervasive role for positive selection in driving the fixation of duplications in *D. simulans* than in *D. melanogaster*. If duplications of complete genes often have only small positive effects on fitness, they will be detected and favored more readily in *D. simulans* than in *D. melanogaster* because of the former's larger effective population size (see Discussion).

There are two other notable differences in the patterns of duplication polymorphism found between *D. simulans* and *D. melanogaster* that could support the hypothesis that both purifying and positive selection are stronger in the *D. simulans*. First, there is a significantly higher proportion of low frequency duplications (i.e. those present in only one of the lines) segregating in *D. simulans* than in *D. melanogaster* (83% vs. 74%, respectively; Fisher's exact test, p = 1.7e^−07^). Because purifying selection is expected to lead to an excess of rare variants, the higher proportion of duplications kept at low frequencies in *D. simulans* could suggest stronger purifying selection. Second, there is an excess of high frequency duplications segregating on the X chromosome when compared to the autosomes in *D. simulans* (Fisher's exact test, p = 0.03) but not in *D. melanogaster* (Fisher's exact test, p = 0.5). Given the different biology and population genetics of the X chromosome, differences found between the X and the autosomes could be due to multiple (and non mutually exclusive) factors [56]. However, if one assumes that most beneficial mutations are recessive or partially recessive, then positive selection is expected to be more efficient on the X than on autosomes (faster X evolution [56]), which would lead to a higher proportion of high-frequency duplications on the X than on autosomes. There is, however, an alternative explanation for both the overall higher proportion of low frequency duplications in *D. simulans*, and the excess of high frequency duplications in the X chromosome of this species. Demographic processes, such as population expansion, bottlenecks and population structure, can also generate these patterns of polymorphism [57]. The two species have different demographic histories, which could easily generate differences in genome-wide patterns of polymorphism between them. Demographic processes cannot, however, explain the differences between the two species in the proportion of coding vs. non-coding duplications for low and high frequency variants. This is because unlike selection, demography cannot discriminate between functional and non-functional regions of the genome, instead affecting equally the genome as a whole.

### Duplication hotspots in *D. simulans* and *D. melanogaster*

Perry and colleagues compared global maps of copy number variation for the human and chimpanzee genomes [28], [30], finding a significant excess of overlap between CNVs of the two species. They proposed that these segments correspond to CNV hotspots, regions of recurrent CNV mutations in both genomes. To examine this question in flies, we compared the distribution of duplications in the genomes of the two *Drosophila* species. Of the 830 duplications detected in *D. simulans*, 769 (93%) were mapped onto the *D. melanogaster* genome (see Materials and Methods). Most of the *D. simulans* duplications that failed to map onto the *D. melanogaster* genome are located close to the pericentromeric regions (which are also regions poorly represented on the tiling arrays due to their repetitive nature). Out of the 769 *D. simulans* duplications mapped onto the *D. melanogaster* genome, 96 (12%) overlap with polymorphic duplications in *D. melanogaster*. Figure S1 shows the location of the overlapping duplications in the genome. The number of duplications that overlap between the two species is significantly higher than what is expected by chance: randomly shuffling the coordinates of *D. simulans* and *D. melanogaster* duplications 1,000 times within each chromosome yielded at most 53 (7%) duplications showing overlap, with a median of 32 (4%) duplications overlapping by chance in the two species (see Materials and Methods).

The clear excess of duplications overlapping between the two *Drosophila* species could be due to either shared ancestral polymorphisms or to recurrent mutation at mutational hotspots. For 67 of 96 overlapping duplications, we can directly exclude the shared ancestral polymorphism hypothesis because the size of the duplicated regions varies considerably between the two species. For the remaining 29 duplications the microarray resolution is insufficient to determine if the breakpoints are the same or not. However, the proposition that these 29 duplications represent ancestral shared polymorphisms is unlikely. Neutral polymorphisms are not expected to be retained for the 2–3 million years that have already passed since these two species split. Only 1% of neutral polymorphisms are expected to be retained after 5.3N generations [58], which assuming a population size (N) of 10^6^ [59] and 10 generations a year, means 99% of shared polymorphisms should be resolved within ∼530,000 years after the two species split. Selection could, in principle, maintain shared polymorphisms for much longer [58] but most of these 29 duplications are either intergenic or intronic, which argues against this hypothesis. Overall, the set of overlapping duplications has a higher fraction of non-coding duplications (i.e. intergenic and intronic duplications) than the general dataset (80% vs. 64%, respectively, Fisher exact test, p = 0.0005). There is no difference in the proportion of partial and complete gene duplications between the two datasets (Fisher exact test, p = 0.3). Table S1 has the location and genomic annotation of all overlapping duplications.

A more likely explanation for the observed excess of overlap between the duplications identified in the two species is that there are orthologous regions in the two *Drosophila* genomes that experience higher rates of duplication. This is also the explanation favored by Perry and colleagues to explain the excess of overlap found between human and chimpanzee CNVs [28], [30].

### Duplication hotspots are associated with late-replicating regions of the genome

CNV hotspot regions shared between human and chimpanzees are strongly enriched with segmental duplications [28], [30]. Segmental duplications are known to facilitate the occurrence of further duplications (and deletions) by mediating non-allelic homologous recombination [3], [4] and are responsible for the high mutation rates observed at some loci associated with genomic disorders [27]. To investigate the causes for the *Drosophila* duplication hotspots, we tested for an enrichment of segmental duplications and transposable elements (also capable of mediating non-allelic homologous recombination) in these regions. We found that the duplications showing overlap between *D. simulans* and *D. melanogaster* were not enriched with either (Fisher exact test, p = 0.7 for segmental duplications and p = 0.9 for transposable elements). Despite the previous observation linking human/chimpanzee segmental duplications with CNV hotspots, this result was not surprising. Segmental duplications are less abundant in fly than in mammalian genomes, and in flies are mainly restricted to pericentromeric regions [60] where none of the duplication hotspots identified here is located (these regions are under-represented in the microarrays because of their repetitive nature). Transposable elements are also mostly kept to pericentromeric regions [61], and those that are not have different distributions in the two *Drosophila* species [46].

In *D. melanogaster*, polymorphic duplications are not distributed uniformly throughout the genome. There are regions of the genome that show unusually high levels of duplication [33]. Importantly, these regions were shown to be significantly associated with regions of the genome that are late-replicating [33]. Hence, we hypothesized that the duplication hotspots identified between the two *Drosophila* species were also associated with these late-replicating regions of the genome. There are several high-resolution replication timing maps available for the *D. melanogaster* genome (e.g. [62], [63]). Here, we use the replication timing profile generated by Schwaiger and colleagues for the *D. melanogaster* Kc cell line, where a Hidden Markov Model was applied to classify the genome into early-, mid- and late-replicating regions [62]. Additional replication timing maps for *D. melanogaster* were generated as part of the modENCODE project for three cell lines (Kc, S2 and Bg3 [63]). Our results were robust to the choice of the replication timing dataset (Figures S2 and S3). Replication timing varies between -4 and 4 with the former indicating late-replicating regions and the latter early-replicating regions.

**Figure 3:**
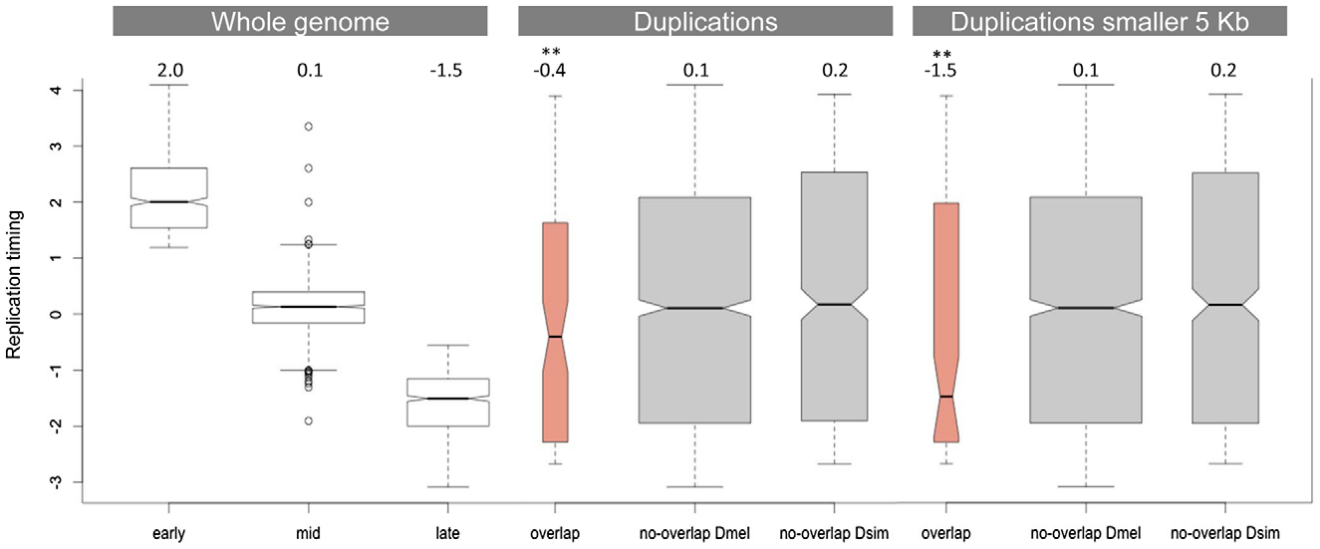
Replication timing of duplications overlapping between *D. simulans* and *D. melanogaster*. The first panel shows the replication timing data for the whole genome as determined by Schwaiger and colleagues [62]. The second panel compares the distribution of replication timing values for duplications that overlap and that do not overlap between the two species. The third panel is similar to the second but considers only duplications smaller than 5 kb (in both species). The numbers on the top of the three panels refer to the observed median replication times. ^**^ indicates a significantly lower replication timing (p<<0.01).

Figure 3 compares the replication timing profile of duplications that do not overlap between *D. simulans* and *D. melanogaster* (grey) and those that do (salmon). Consistent with the observation that regions of the *D. melanogaster* genome that are rich in duplications tend to be late-replicating [33], we found that duplications that overlap between the two species are also significantly enriched in late-replicating regions (Wilcoxon rank sum test, p = 0.002). This result is strengthened if we restrict our analysis to those duplications that are smaller than 5 kb and therefore are less likely to show overlap due to chance alone. In this latter case, the median replication timing observed for duplications that overlap between the two species is -1.5 (p = 0.001), which is also the median replication timing observed for late-replicating regions of the genome as a whole. Figures S2 and S3 show this same analysis using the replication timing profiles of the three cell lines generated as part of the modENCODE project (Kc, S2 and Bg3 cell lines). The results are qualitatively similar.

Late-replicating regions of the genome have lower gene density than early- and mid-replicating regions, which means they have larger intergenic regions. We also determined that genes located in late-replicating regions have longer introns than genes located in early- and mid-replicating regions (median of 200 bp, 77 bp and 88 bp, respectively). Given that we observe strong purifying selection against duplications encompassing coding regions, the association between duplications that overlap between the two species and late-replicating regions could be due to a higher proportion of non-coding sequences in these late-replicating regions. Three independent observations do not support this possibility. First, there is not an overall increase of *D. simulans* duplications in late-replicating regions as would be expected if they were accumulating in these regions due to a lower selective constraint. However, there is a significant excess of overlapping duplications in late-replicating regions when compared to non-overlapping duplications (Fisher exact test, p = 0.009). The same holds true for *D. melanogaster* duplications. Although there is an overall excess of *D. melanogaster* duplications in late-replicating regions (binomial test, p = 0.028), the proportion of overlapping duplications in late-replicating regions is significantly higher than the proportion of non-overlapping duplications (Fisher's exact test, p = 0.03). Second, we compared the observed number of late-replicating duplications that overlap between the two species with what would be expected by chance alone. Although there are 47 duplications located in late-replicating regions that overlap between the two species, when we shuffled the coordinates of duplications in late-replicating regions 1,000 times exclusively within late-replicating regions we observed at most 12 duplications showing overlap, with a median of 4 (i.e. only 9% of the actual observed number of duplications showing overlap). Third, late-replicating duplications do not show evidence of lower constraint in their site-frequency spectra when compared to duplications located in either early- or mid-replicating regions (as measured by comparing the proportion of duplications segregating in only one line). We therefore conclude that selection is not responsible either for the excess of overlap found between duplications in *D. melanogaster* and *D. simulans* or for the enrichment of these duplications in late-replicating regions. Instead our data provides strong evidence for the hypothesis proposed previously [33] that replication timing impacts the genomic distribution of duplication rates. Our data further suggests that the existence of duplication hotspots within late-replicating regions is not simply a consequence of the accumulation of duplications in these regions. Late-replicating regions are probably acting synergistically with other factors, such as particular types of sequences (e.g. more prone to breakage) or higher-order chromatin features (e.g. chromatin condensation), to generate the duplication hotspots. Hence, late-replicating regions do not act homogeneously as duplication hotspots. Instead, duplication hotspots correspond to discrete regions that tend to be located within late-replicating regions.

## Discussion

### Duplication hotspots are enriched in late-replicating regions of the genome

The density of duplications has been shown to vary throughout the human [26], [27] and fly genomes [33], and the existence of duplication hotspots has been suggested for these and other species [28], [31]. By comparing the distribution of polymorphic duplications along two *Drosophila* genomes, we found a significant excess of duplications overlapping between the two species, suggesting the existence of shared duplication hotspots. In mammalian genomes duplication hotspots are associated with genome regions enriched in segmental duplications [27], [28], [31]. We did not find an enrichment of these sequences in *Drosophila* duplications hotspots. Rather, we found that duplication hotspots are significantly associated with late-replicating regions of the genome, further supporting the hypothesis that some regions within late-replicating regions of the genome experience increased rates of duplication [33].

Prior observations support a link between replication timing and the formation of duplications. For example, in yeast, large spontaneous duplications are associated with replication termination sites [64]. Fragile sites in both humans and *Drosophila* have been proposed to represent sequences that are late-replicating [65]–[67] and, at least in humans, fragile sites are hotspots for chromosomal rearrangements in cancer [65], [68] and are also likely to mediate structural variation in the germline [65]. A recent study [69] suggested that fragile sites occur in regions of the genome showing a paucity of replication initiation events. Sparseness of initiation sites would force replication forks to cover longer distances to finish replication, thereby creating the association between fragile sites and late-replication. We tested this hypothesis by determining whether duplications overlapping between the two *Drosophila* species tended to be, on average, located further away from known origins of replication than the remaining duplications (and a randomly generated set of sequences). We found no significant difference between the two sets of duplications in their distance to known origins of replication (for origins of replication identified in the Kc, S2 and Bg3 cell lines as part of the modENCODE project (data not shown [63])). It is important to note, however, that the location of origins of replication (and replication timing) can vary among cell types [62], [65], [69] and all analysis reported here were conducted using data obtained from cell lines instead of germline cells.

Because replication-associated repair is proposed to be responsible for the formation of both simple and complex CNVs in the human genome [20]–[22], [24], [70], the presence of *Drosophila* duplication hotspots in late-replicating regions of the genome could be interpreted as supporting an important role for replication-associated repair in the formation of CNVs in these species. However, the association between late-replicating regions and duplication hotspots does not necessarily imply that the latter arise as a direct consequence of replication-associated repair. An increase in DNA double-strand breaks and/or stalled replication forks in particular regions within late-replicating regions that are (incorrectly) repaired by the canonical DNA repair pathways (i.e. non-homologous end-joining or homologous recombination) would also generate this association. Similarly, duplication hotspots could be associated with regions within late-replicating regions that, while experiencing normal rates of DNA double strand breaks, have a higher rate of incorrect repair (for example, because of higher chromatin condensation).

It is also important to note that in *D. melanogaster*, only duplication-rich regions of the genome were found to be associated with late-replicating regions. Deletion-rich regions were associated instead with early-replicating regions [33]. This is in apparent contradiction with the concept that fragile sites are associated with duplication hotspots because fragile sites are expected to be associated with both types of rearrangements, not only with duplications [65], [68]. However, deletions tend to be more deleterious than duplications [36], [37] and so purifying selection preferentially removes them from the population. As a result, even though similar numbers of deletions and duplications are created at hotspots, because of stronger purifying selection acting on deletions, an excess of duplications is instead observed. The existence in *D. melanogaster*'s early-replicating regions of deletion-rich regions, but not of duplication-rich regions, can be explained by the fact that non-homologous end joining is the preferred repair pathway in early S phase (i.e. in early-replicating regions) and that it mostly creates deletions [3], [4], [18]. In late S phase (i.e. in late-replicating regions) homologous recombination is the preferred DNA repair pathway and it generates both duplications and deletions [3], [4], [18].

Because our work suggests that duplication hotspots are enriched within late-replicating regions of the genome, we asked if there are particular classes of genes enriched in these regions. A Gene Ontology analysis of the genes located in late-replicating regions of the *D. melanogaster* genome revealed that these regions are significantly enriched with sensory genes, both olfactory genes (Holm-Bonferroni correction, p = 5.3×10^−5^) and gustatory genes (Holm-Bonferroni correction, p = 2.3×10^−4^). We confirmed this result by determining the replication timing for all olfactory receptor and gustatory receptor genes (as defined by [71]). Although only ∼20% of the genes in the genome are located in late-replicating regions, more than 40% of gustatory receptor genes and more than 50% of olfactory receptor genes are late-replicating. Figure S4 compares the distribution of replication timing of olfactory receptor and gustatory receptor genes and all genes in the genome. Both classes of sensory genes tend to be late-replicating (p = 0.003 for gustatory receptor genes and p = 2.5×10^−8^ for olfactory receptor genes), but olfactory receptor genes tend to replicate later than gustatory receptor genes (median replication timing of 0.21 and -1.6, respectively). In the set of *D. melanogaster* duplications, 5 overlap with olfactory receptor genes and 4 with gustatory receptor genes and in the set of *D. simulans* duplications, 2 overlap with olfactory receptor genes. There is further evidence in the literature of copy number variation in sensory genes in *D. melanogaster* (e.g. [34], [72]). The number of observed duplication polymorphisms encompassing sensory genes is, however, likely to be an under-estimation of the actual number of duplication polymorphisms associated with this class of genes. Microarray probes have to map to unique regions of the genome, which excludes regions with recent gene duplications, such as some of the regions that harbor sensory genes. For this reason, the abundance of sensory genes among copy number variants in *Drosophila* should be re-examined using next generation sequencing technology, which should not be affected by the existence of recent duplicates (for an encouraging first step see [34]).

What would be the predicted dynamics of sensory genes in *Drosophila*? Our data suggests that duplication hotspots are enriched within late-replicating regions, but that does not mean that sensory genes are enriched in the late-replicating regions *hotspots*. Additionally, our data suggests an important role for selection in the fixation of duplications in *Drosophila*. Thus, even if sensory genes experience, on average, higher duplication rates, this may not necessarily translate into increased numbers of fixed differences in the number of sensory genes between species. Accordingly, McBride and Arguello found little variation in the number of olfactory and gustatory genes in the *D. melanogaster* subgroup of species (the exception being a high rate of loss of gustatory receptor genes in the two *Drosophila* specialists: *D. sechellia* and *D. erecta* [71] caused by nonsense mutations, not by deletions). High rates of duplication for sensory genes would predict instead increased levels of within-species duplication polymorphisms, which could be translated into increased levels of variation in gene expression. Testing this hypothesis awaits an appropriate dataset describing population-level variability in levels of gene expression for olfactory and gustatory receptor genes in either of the two *Drosophila* species.

### Duplication polymorphisms are under strong selection in *D. simulans*

Several lines of evidence suggest that selection plays a major role in shaping patterns of duplication polymorphism in *D. simulans*. The action of purifying selection can be seen in the skew of duplications toward low frequency variants (i.e. 83% of duplications are present in only 1 of the 14 lines) but more robustly (with regards to the alternative hypothesis of demography) in the strong depletion of coding duplications. A role for positive selection can also be inferred. There is a significant over-representation among high-frequency duplications (segregating in at least 6 of the 14 lines), of complete gene duplications (35% of all high-frequency duplications). Although there are many ways in which duplications can generate novel phenotypes (e.g. [15], [16], a large fraction are expected to be complete gene duplications [11], [13], like the ones segregating at high-frequency in *D. simulans*.

A comparison of the patterns of duplication polymorphism between *D. simulans* and *D. melanogaster* suggests stronger selection in the former. The dearth of duplicates overlapping coding sequence is significantly stronger in *D. simulans* than *D. melanogaster*, as is the skew of duplications toward low frequency variants. While this latter difference can also be explained by the different demographic histories of the two species (and of the lines used for each species), the difference in the duplication density in coding sequence can only be explained by stronger purifying selection acting on *D. simulans* duplications. On the other side of the frequency spectrum, positive selection also seems stronger in *D. simulans*. Although in *D. melanogaster* there is also a significant increase in the proportion of complete gene duplications among those duplications segregating at high-frequency, there is a significantly higher proportion of complete gene duplications segregating among high-frequency duplications in *D. simulans* than in *D. melanogaster*. The hypothesis of stronger selection in *D. simulans* than *D. melanogaster* is consistent with previous data suggesting that *D. melanogaster* has experienced a reduction in its effective population size [49], [50]. Because the effectiveness of selection is determined by the product of the effective population size and the intensity of selection [73], the larger the effective population size, the more effective both purifying and positive selection are expected to be. Several observations support this notion for the two *Drosophila* species. For example, *D. simulans* has a higher codon bias than *D. melanogaster* [74], there are higher levels of amino acid polymorphism in *D. melanogaster* than *D. simulans* [49] and there are stronger signatures of purifying selection at synonymous sites in *D. simulans* than *D. melanogaster* [51].

Most population genetic models that attempt to describe the early evolutionary trajectories of new duplications (i.e. gene duplications) assume that the force responsible for the fixation of the duplication is genetic drift [43], [44]. These models assume that the ultimate fate of the duplication is dictated by subsequent mutations that occur in one or both copies, which can lead to the permanent preservation of the duplication in the genome or, alternatively, allow its loss [14], [43], [44]. *D. simulans*' duplication polymorphism data suggests instead an important role for selection in the fixation of a significant fraction of duplications. A study of a small number of recently fixed gene duplications in the *Arabidopsis thaliana* genome also suggested an important role for positive selection in driving these variants to fixation [75]. If the observation made here for *D. simulans*, that selection plays an important role in the fixation of duplications, holds true, then population genetic models will have to include positive selection when modeling the early stages of the evolution of this class of mutations (for an example see [76]). The observation that a large fraction of duplications are fixed not by drift but by positive selection should not be surprising in light of the overwhelming evidence that between 40-50% of amino acid substitutions in *Drosophila* species are adaptive [77].

## Materials and Methods

### Identification of *D. simulans* polymorphic duplications

We generated the dataset of *D. simulans* duplications by hybridizing the DNA of 14 natural lines to Affymetrix *D. melanogaster* tiling arrays (three replicates per line). Each tiling array was hybridized with DNA pooled from 30 female virgin flies. Among the 14 lines, 9 were from three different locations in Madagascar (MD01, MD04, MD72, MD105, MD197, MD210, MD222, MD236 and MD239), one was from Israel (SFSR2IIST), one from Reunion Island (W74), one from New Guinea, one from Kenya (Impala 6) and one from Indiana (Valparaiso). The *D. simulans* lines were selected with the goal of maximizing levels of variability and so were mostly sampled from the known diversity center of the species [46]. The protocol used to prepare the DNA samples for the microarray experiments was the same one used by Emerson and colleagues to detect CNVs in the *D. melanogaster* genome [36].

The hybridization intensities were decoded into differences in copy number using a Hidden Markov Model. The Hidden Markov Model used here is the same one used by Emerson and colleagues to detect CNVs in the *D. melanogaster* genome [36]. The only difference between the two genomic surveys lied in the number of probes used. Since we only wanted to use those probes in the array that had a unique and perfect match to the *D. simulans* genome, we used MegaBlast to blast all ∼3,000,000 probes present in the array against this genome (droSim1) and kept only those that met our criteria [78]. We ended up with ∼900,000 probes with which to survey the *D. simulans* genome. The raw microarray data and the results from the Hidden Markov Model are deposited in GEO under the accession (GSE29260).

We classified *D. simulans* duplications as intergenic if they encompass exclusively intergenic sequence, as intronic if they encompass exclusively intronic sequence, as a partial gene duplication if they encompass exonic sequence or exonic and intronic sequence, and finally as a complete gene duplication if they encompass the complete gene structure of a gene (protein-coding or non-protein-coding). Table S1 contains the location of each duplication and its annotation. We looked for the presence of noncoding genes within our dataset using the current *D. simulans* genome annotation. There is only one non-protein coding gene that overlaps with one duplication: a small nucleolar RNA (snoRNA, FBgn0256493), completely duplicated and present in 1 of the 14 lines. In *D. melanogaster* there are 11 duplications that overlap with noncoding genes. We used BEDTools (v2.10.1) [79] to compare the coordinates of the duplications with the genomic coordinates of all gene structures annotated as part of the Release 3.1 of the *D. simulans* genome.

### Evaluation of the quality of the *D. simulans* duplication calls

We evaluated the quality of the duplication calls by attempting to confirm a subset of 24 by PCR (and long-range PCR). We used two different strategies. The first was to design a pair of divergent primers within the predicted boundaries of the duplication so that there would only be DNA amplification in the presence of a tandem duplication. Using this strategy we confirmed 18 duplications. Some of the duplications required long-range PCR instead of regular PCR because the amplified bands were larger than 5 kb. We performed long-range PCR using the TaKaRa La Taq system and the recommended protocol. The second strategy was to design a pair of convergent primers outside the predicted duplication boundaries. The presence of a tandem duplication creates a band larger than expected. This second strategy required the use of long-range PCRs and confirmed one additional duplication. We sequenced some of the duplication breakpoints identified using the first strategy. There was a good agreement between the predicted and the actual breakpoints (Figure S5). The final PCR validation rates for the *D. melanogaster* and *D. simulans* duplications were 64/74 (86%) [36] and 19/24 (79%) respectively, and were not significantly different from each other (Fisher's exact test, p = 0.86).

The strategy of designing divergent pairs of primers within the putative duplications imposed a limit on the size of the duplications assayed. We limited our confirmations to duplications larger than 300 bp. The confirmation dataset has a mean size of 2.6 kb (vs. 1.8 kb in the general dataset) and the smallest duplication confirmed was 332 bp. The duplications present in the *D. melanogaster* confirmation dataset were, on average, 5 kb [36]. The duplications in the confirmation dataset were chosen blindly regarding their posterior probabilities of duplication (the output of the Hidden Markov Model) and number of probes suggesting the duplication (the smallest duplication was covered by 5 probes). There were no differences between the confirmation dataset and the general dataset in terms of frequency (i.e. the proportion of duplications detected in only one line vs. multiple lines) and genomic annotation. Included in the confirmations are 3 *D. simulans* duplications showing overlap. Within the confirmation dataset there were no apparent differences between the set of duplications confirmed and those that were not. However, given that only 5 duplications were not confirmed there would be little power to detect any differences, even if they existed. Table S1 has the location (and characterization) of the duplications confirmed and those not confirmed.

### Modifications to the set of *D. melanogaster* duplications

Although for a duplication to be called in *D. simulans*, two consecutive probes had to have hybridization intensities decoded by the Hidden Markov Model as being duplicated, in the original *D. melanogaster* dataset only one probe was required. Thus, we removed from the set of *D. melanogaster* duplications all those that were predicted by only one probe. This resulted in excluding 195 duplications. We also converted the *D. melanogaster* duplication coordinates from release 4 to release 5 using FlyBase's coordinate converter (http://flybase.org/static_pages/downloads/COORD.html), and updated the genome annotation to release R5.33.

### Mapping the set of *D. simulans* duplications onto the *D. melanogaster* genome

We mapped the duplications identified in *D. simulans* onto the *D. melanogaster* genome (release 5) with BLAT [80] by selecting the reciprocal best hit between the two genomes. Of the 830 duplications, 769 were unequivocally mapped. Most duplications that failed to map were located close to pericentromeric regions in *D. simulans* and either had no good hit in *D. melanogaster* or mapped to multiple locations. We required at least 90% of the region duplicated in *D. simulans* to be unambiguously mapped to the *D. melanogaster* genome and the difference between the region duplicated in *D. simulans* and its ortholog in *D. melanogaster* not to exceed 30% of the size of the duplication in *D. simulans*.

### Determining the significance of the overlap observed between *D. simulans* and *D. melanogaster* duplications

Duplications were considered to overlap when at least 1 bp of a duplication in *D. simulans* overlapped with 1 bp of a duplication in *D. melanogaster*. In order to evaluate the significance of the observed number of duplications that overlap between the two species, we compared it with what was observed for 1,000 sets of randomly generated coordinates created using BEDTools (i.e. BEDshuffle) [79]. For each species, we generated 1,000 datasets, perfectly matching the duplication datasets by shuffling the coordinates within each chromosome. Then, for each of the 1,000 datasets in each species we determined their overlap. We also did a similar analysis focusing only on late-replication regions. For this analysis we generated 1,000 matching sets for the duplications located in late-replicating regions and shuffled the coordinates exclusively within these regions.

### Association between duplication hotspots and segmental duplications/transposable elements

We used the map of segmental duplications identified by Fiston-Lavier and colleagues [60] and the map of transposable elements identified by Bergman and colleagues [61] for the *D. melanogaster* genome to evaluate the association between these elements and duplications overlapping between the two species. We considered a duplication to be associated with either a segmental duplication or a transposable element if the distance between them was smaller than 2 kb (including direct overlap with the duplication). For both datasets we updated the coordinates from release 4 to release 5 using the tool Coordinate Converter on Flybase.

### Replication timing of duplication hotspots

Schwaiger and colleagues [62] generated the replication timing data described along the text. They generated five replication timing profiles for Kc cells using Affymetrix tiling arrays, which were averaged and then smoothed to generate the replication timing profile for this cell line. Then, using a Hidden Markov Model, they classified genomic regions into early-, mid- and late-replicating [62]. The other replication timing datasets (for Kc, Bg3 and S2 cell lines) were downloaded directly from the modENCODE webpage (http://www.modencode.org/). A very small number of duplications overlapped with more than one replication timing environment (e.g. early- and mid-replicating regions). For these duplications, the replication timing corresponded to the mean replication timing of the two environments.

In order to determine if duplications showing overlap between the two species are located, on average, further away from origins of replication than the remaining duplications, we calculated the distance between the two sets of duplications to the origins of replication identified in the Kc, Bg3 and S2 cell lines as part of the modENCODE project (data downloaded directly from the modENCODE webpage). We also compared these results with the distribution of median distances to the three sets of origins of replication generated for 1,000 random sets of coordinates matching the duplication datasets.

### Enrichment of olfactory and gustatory receptor genes in late-replicating regions

In order to determine if there are particular classes of genes enriched in late-replicating regions we first classified all genes in the genome as early-, mid- or late-replicating. Some genes overlapped with more than one replication timing environment. For these genes we selected the replication timing environment closest to the start of the gene. The results did not change if we chose instead the replication timing environment closest to the end of the gene or if we excluded genes overlapping more than one replication timing environment. We used the Gene Ontology tool on FlyMine [81] (using the Holm-Bonferroni correction for multiple testing) to see if there were any classes of genes enriched in the set of genes classified as late-replicating. We performed this same analysis using the Gene Ontology tool Gorilla [82], which gave similar results (i.e. enrichment in olfactory and gustatory genes).

We used the complete list of olfactory and gustatory receptor genes identified by McBride and Arguello [71] to ascribe for each gene their replication timing. If a gene overlapped with more than one replication timing environment we ascribed that gene the mean replication time for the two environments. We also used this list of genes and the list of olfactory and gustatory receptor genes identified in the *D. simulans* genome by the same authors to identify the polymorphic duplications in both *Drosophila* species encompassing these genes.

All statistical analyses were done using the statistical package R [83] and the application Rstudio (http://www.rstudio.org/).

## Supporting Information

Figure S1 Genomic distribution of duplications in *D. simulans* (filled bar chart), *D. melanogaster* (open bar chart) and duplication hotspots (arrows). This figure was generated using the Karyotype tool in *D. melanogaster*'s Ensemble webpage (http://metazoa.ensembl.org/Drosophila_melanogaster/Location/Genome).
(TIF)

Figure S2 Comparison of the replication timing of duplications overlapping and non-overlapping between *D. simulans* and *D. melanogaster* for the modENCODE data. The p-values are the result of a Wilcoxon rank sum test comparing replication timing of duplications in *D. simulans* that overlap and that do not overlap with duplications in *D. melanogaster*.
(TIF)

Figure S3 Comparison of the replication timing of duplications, smaller than 5 kb, overlapping and non-overlapping between *D. simulans* and *D. melanogaster* for the modENCODE data. The p-values are the result of a Wilcoxon rank sum test comparing replication timing of duplications in *D. simulans* that overlap and that do not overlap with duplications in *D. melanogaster*.
(TIF)

Figure S4 Replication timing of olfactory and gustatory genes. A. Replication timing data was retrieved from Schwaiger and colleagues [62] and the list of sensory genes from McBride and Arguello [71]. The values refer to the median replication timing values and the p-values are the result of a Wilcoxon rank sum test comparing the distributions of replication timing values for all genes in the genome versus gustatory and olfactory receptor genes. B. Proportion of genes that are early-, mid- and late-replicating. The numbers refer to the number of genes in each class.
(TIF)

Figure S5 Predicted versus real duplication breakpoints. The graph shows the posterior probability of each probe being duplicated, the green bars the predicted breakpoints for the duplication, and the red line the actual limits of the duplication (obtained through Sanger sequencing).
(TIF)

Table S1 Location and annotation of all duplications detected in *D. simulans*. The table contains for each duplication identified in the *D. simulans* genome its genomic location (columns A-C), size (D), frequency in the 14 lines (E), annotation (F), number of genes affected (G), whether or not they were confirmed (H), whether or not they were correctly mapped to the *D. melanogaster* genome (I), and whether or not they overlap with duplications in *D. melanogaster* (J).
(XLSX)
