# Supplementary figures and images for: Drosophila Duplication Hotspots Are Associated with Late-Replicating Regions of the Genome
Source: PLoS Genet. 2011 Nov 3;7(11):e1002340. doi: 10.1371/journal.pgen.1002340 (PMC3207856; doi:10.1371/journal.pgen.1002340)

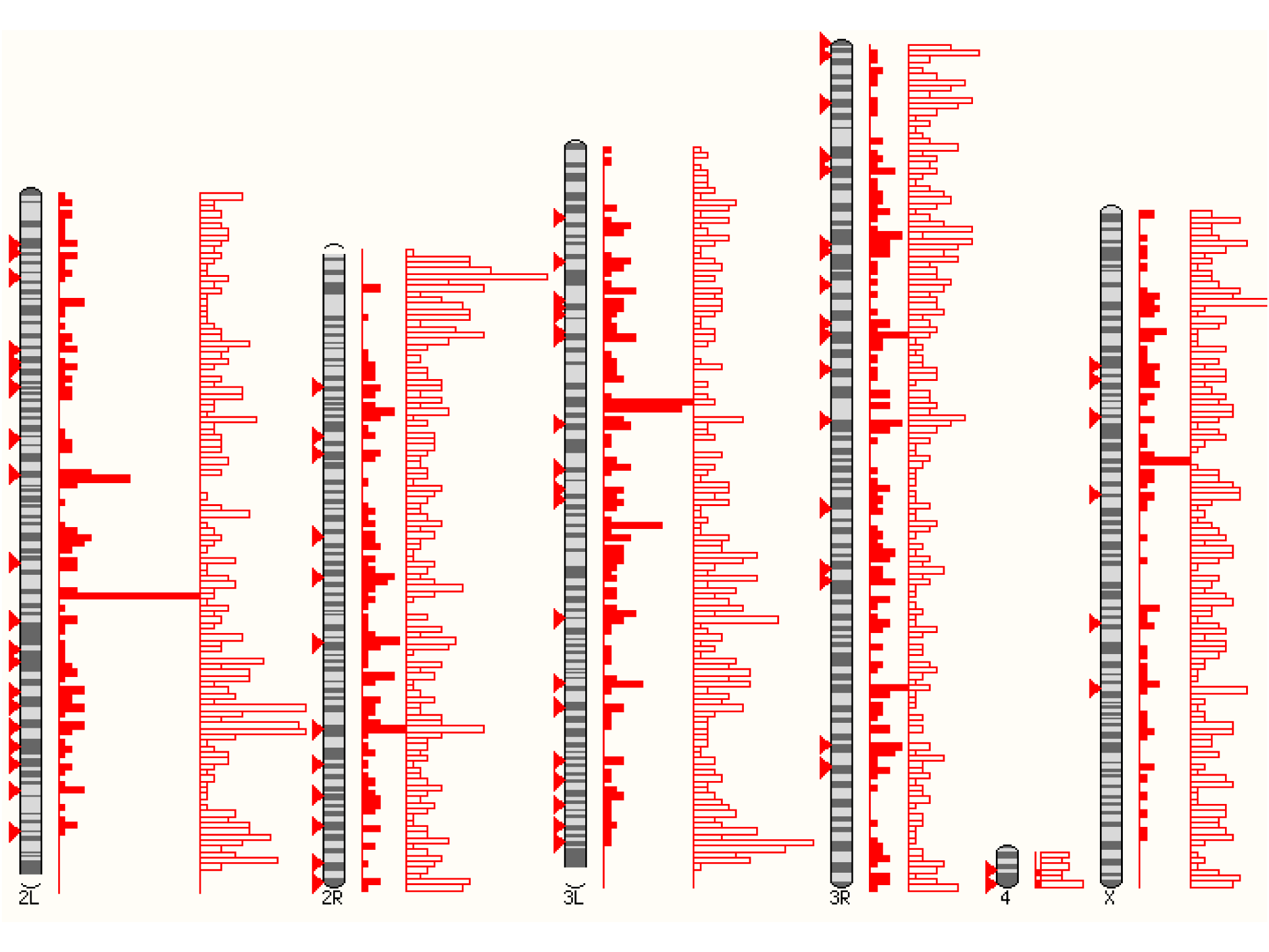

Supplement: Figure S1 — Genomic distribution of duplications in D. simulans (filled bar chart), D. melanogaster (open bar chart) and duplication hotspots (arrows). This figure was generated using the Karyotype tool in D. melanogaster's Ensemble webpage (http://metazoa.ensembl.org/Drosophila_melanogaster/Location/Genome). (TIF) [file pgen.1002340.s001.tif]

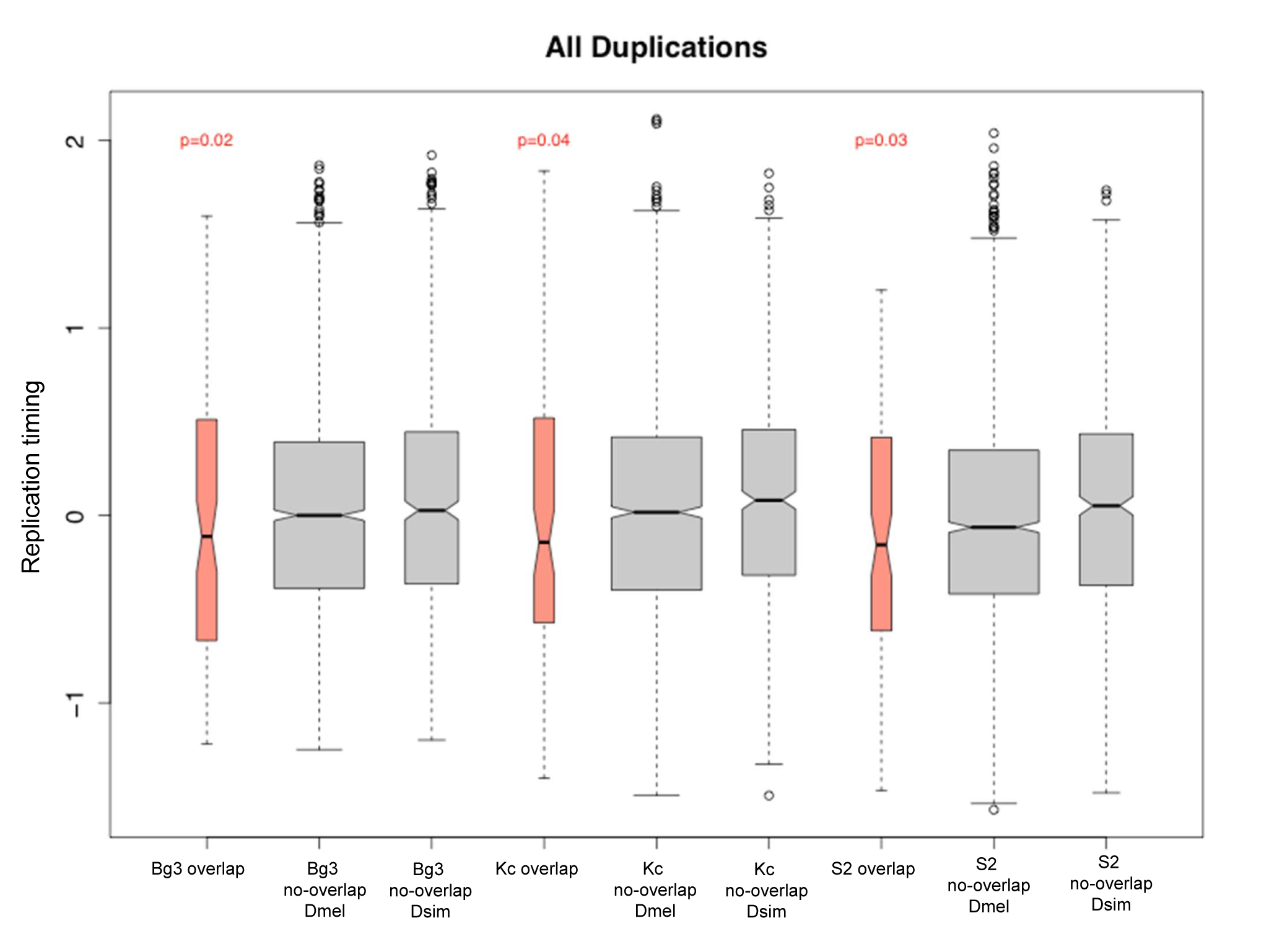

Supplement: Figure S2 — Comparison of the replication timing of duplications overlapping and non-overlapping between D. simulans and D. melanogaster for the modENCODE data. The p-values are the result of a Wilcoxon rank sum test comparing replication timing of duplications in D. simulans that overlap and that do not overlap with duplications in D. melanogaster. (TIF) [file pgen.1002340.s002.tif]

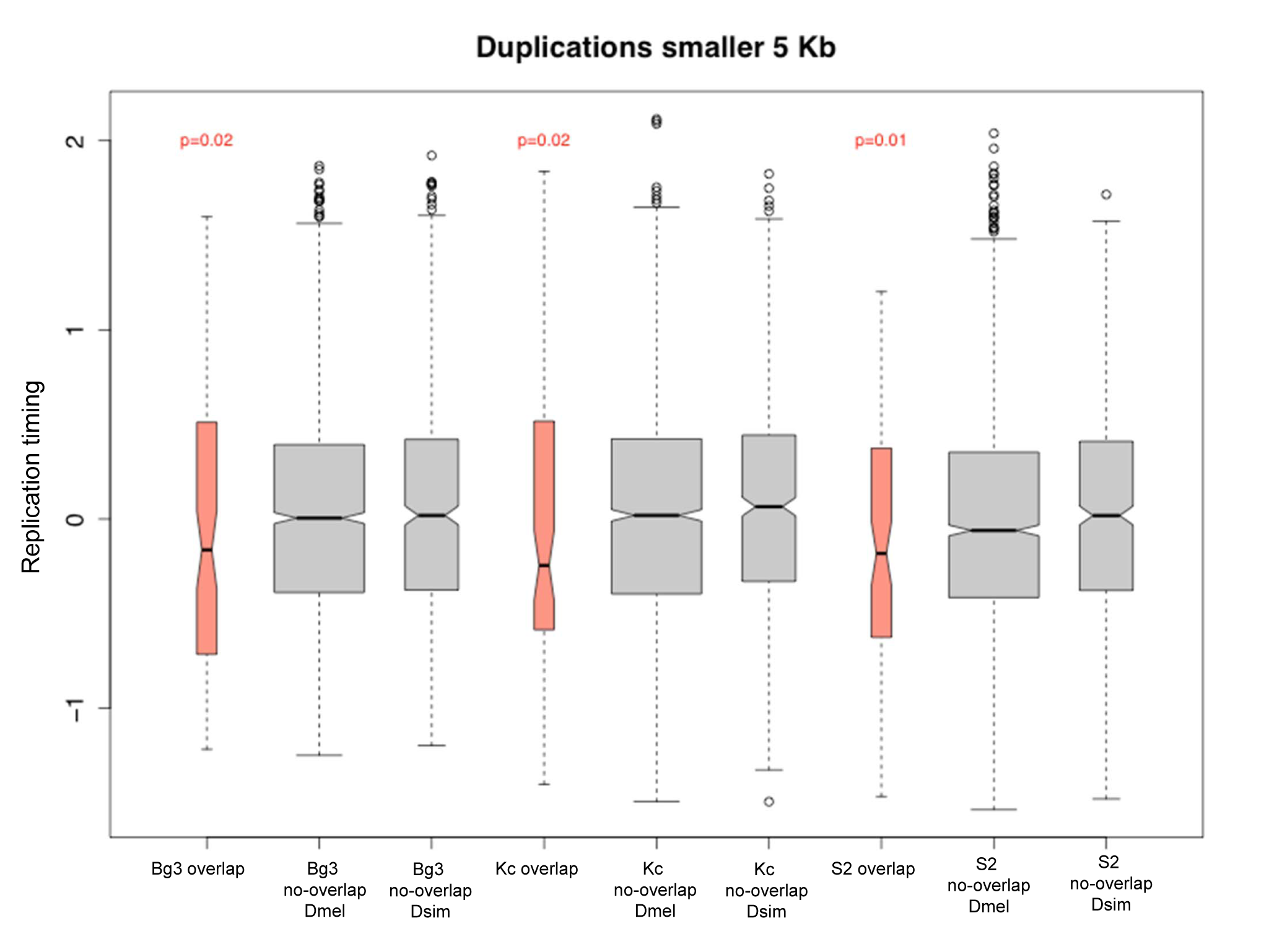

Supplement: Figure S3 — Comparison of the replication timing of duplications, smaller than 5 kb, overlapping and non-overlapping between D. simulans and D. melanogaster for the modENCODE data. The p-values are the result of a Wilcoxon rank sum test comparing replication timing of duplications in D. simulans that overlap and that do not overlap with duplications in D. melanogaster. (TIF) [file pgen.1002340.s003.tif]

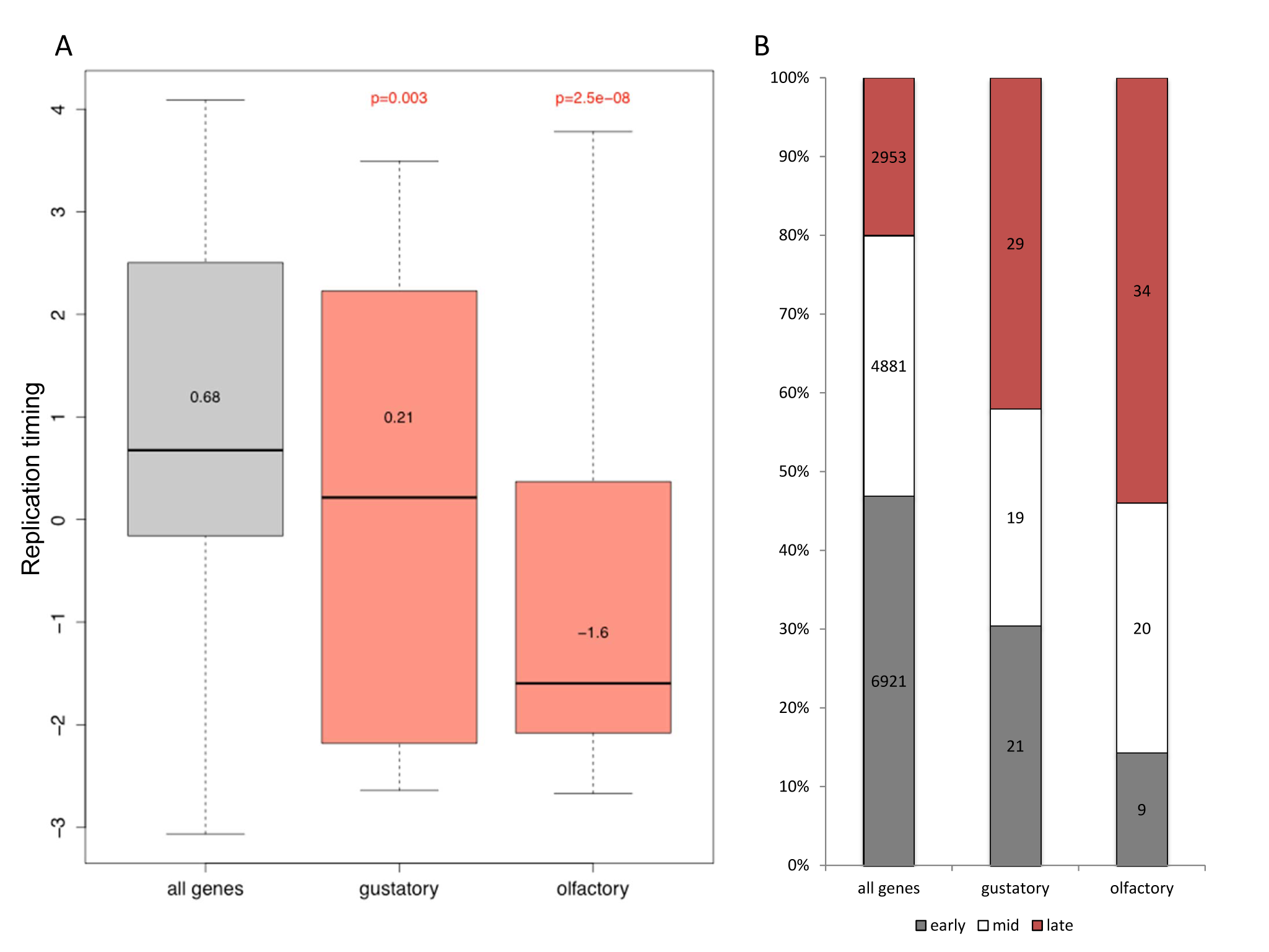

Supplement: Figure S4 — Replication timing of olfactory and gustatory genes. A. Replication timing data was retrieved from Schwaiger and colleagues [62] and the list of sensory genes from McBride and Arguello [71]. The values refer to the median replication timing values and the p-values are the result of a Wilcoxon rank sum test comparing the distributions of replication timing values for all genes in the genome versus gustatory and olfactory receptor genes. B. Proportion of genes that are early-, mid- and late-replicating. The numbers refer to the number of genes in each class. (TIF) [file pgen.1002340.s004.tif]

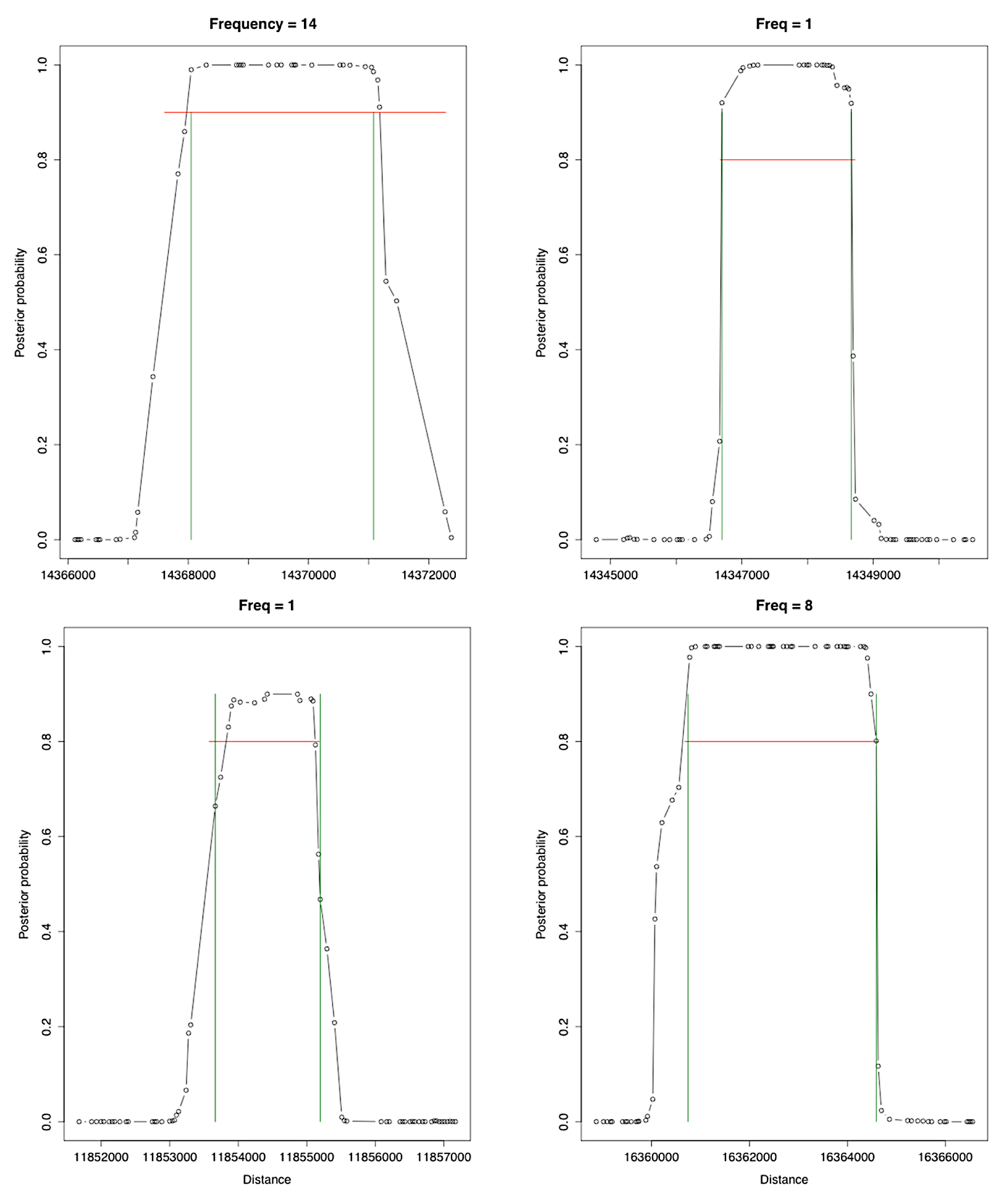

Supplement: Figure S5 — Predicted versus real duplication breakpoints. The graph shows the posterior probability of each probe being duplicated, the green bars the predicted breakpoints for the duplication, and the red line the actual limits of the duplication (obtained through Sanger sequencing). (TIF) [file pgen.1002340.s005.tif]
